# Amyl Nitrite-Induced Hemolytic Anemia: Acute Therapy and Prevention

**DOI:** 10.7759/cureus.16099

**Published:** 2021-07-01

**Authors:** Amira H Elshikh, Ghazal Kango, Marwa Baalbaki, Jeffrey Lankowsky, Amandeep Bawa

**Affiliations:** 1 Internal Medicine, George Washington University, Washington, USA; 2 Pulmonology and Critical Care, George Washington University, Washington, USA; 3 Pulmonary and Critical Care Medicine, Veterans Affairs Medical Center, Washington DC, USA

**Keywords:** acute hemolytic anemia, methemoglobinemia, inhaled nitrites, substance recreational use, msm, provider awareness

## Abstract

Inhaled nitrites have been a substance of recreational use for centuries, primarily among men who have sex with men (MSM). However, there is a lack of awareness of the use of inhaled nitrites in this population and the possible complications and health disparities it carries. In this case report, we present a 62-year-old man with a past medical history of glucose-6 phosphate dehydrogenase (G6PD) deficiency presenting with severe hemolytic anemia and methemoglobinemia after recreational use of inhaled nitrites. The case was complicated with the presence of methemoglobinemia in a patient with G6PD deficiency. This report also aims to increase awareness of the use of nitrites in the MSM population. The use of nitrites is a predictor for high-risk sexual behavior and is associated with positive human immunodeficiency virus (HIV) status.

## Introduction

Inhaled nitrites, referred to as “poppers,” are substances of recreational use tracing as far back as the nineteenth century when they were used to heighten stimulation and induce muscle relaxation [1]. Anal sphincter relaxation and the facilitation of penile erection rendered inhaled nitrites a popular drug amongst men who have sex with men (MSM) [1]. Nitrites can also act as potent oxidants leading to functional anemia by inducing methemoglobinemia through oxidizing hemoglobin from ferrous to ferric form, hindering it incapable of carrying oxygen [1]. Furthermore, hemolysis can occur due to oxidative stress [2]. Although this is a reported complication, the frequency and morbidity of symptomatic methemoglobinemia with inhaled nitrites are poorly documented. In one case series review, 25 cases of methemoglobinemia associated with inhaled nitrates were assessed with three mortalities suspected to be from cardiac arrest [1]. This complication has been seen more so with patients with underlying anemias, such as glucose-6 phosphate dehydrogenase (G6PD) deficiency, the most common genetic deficiency in the world [2]. G6PD deficiency predisposes affected patients to develop acute hemolytic anemia with the administration of oxidative agents. This is due to the function of G6PD and its importance in the initial step in the pentose-phosphate pathway of glycolysis with the production of glutathione [3]. Although some preparations of inhaled nitrites (e.g. “Rush”) are known to cause hemolysis in G6PD-deficient patients, not all of these volatile nitrites are absolutely contraindicated in this population [4]. Much of this is due to the variation of regulation of nitrite compounds globally despite evidence that inhaled nitrites have become more prominent for sexual recreational use since the turn of the century [5]. A study from Germany found that 35% of men who identify as homosexual or bisexual reported the lifetime use of recreational inhaled nitrates [6]. Prior studies have shown that the use of recreational inhaled nitrites among men who identify as homosexual or bisexual was associated with an increased risk of unsafe sexual encounters and higher rates of human immunodeficiency virus (HIV) [6-8]. This case report highlights the complications seen with inhaled nitrites in the MSM community, the challenge it poses with therapy in the G6PD-deficient population, and to increase provider awareness of inhaled nitrite use and its possible consequences.

## Case presentation

A 62-year-old man, with a reported history of G6PD deficiency, coronary artery disease status post-drug-eluting stent on clopidogrel, congestive heart failure with reduced ejection fraction, HIV, and end-stage renal disease on hemodialysis, presented to the emergency department after developing 30 minutes of atypical chest pain associated with one-time emesis. He admitted to using recreational “poppers” (inhaled amyl nitrite) for the first time prior to presentation but denied other substance abuse or medication changes. On arrival, the patient was hypotensive, with tachypnea and hypoxemia requiring supplemental oxygen. His exam was concerning for dry mucous membranes, scleral icterus, and severe diffuse jaundice. He was admitted to the intensive care unit where blood work showed hemoglobin of 7.2 g/dL (decreased from his baseline of 10.5 g/dL). He had thrombocytopenia, which was stable from baseline. Total bilirubin was 10.5 mg/dl with a direct bilirubin component of 4.6 mg/dl, and alkaline phosphatase was 179 mU/ml. Initial lactic acid was 3.7 mmol/L. An arterial blood gas (ABG) was delayed by several hours but eventually showed a pH of 7.494, partial pressure of carbon dioxide (pCO2) 28.9 mmHg, partial pressure of oxygen (pO2) 72.5 mmHg on fraction of inspired oxygen (Fio2) of 28%, a lactic acid level that increased to 8.3 mmol/L, and a methemoglobin level of 1.5%. A peripheral blood smear showed anisopoikilocytosis red blood cells (RBCs) with Heinz bodies as well as a few schistocytes. He was transfused one unit of packed RBCs with an appropriate response to 8.1 g/dL. Hemolysis was confirmed by the presence of elevated retic percent of 6%, reticulocyte count of 144 K/mm^3^, and total bilirubin of 14 mg/dl. D-dimer was normal at 3.85 mcg/mL, fibrinogen mildly elevated at 316 mg/dl, partial thromboplastin time was 37.7 seconds, prothrombin time was 20.2 seconds, International normalized ratio (INR) was 1.7, haptoglobin was <8 mg/dL, and moderately elevated lactate dehydrogenase (LDH) at 524 U/L. A direct Coombs test was negative. The urine drug screen was negative. Computed tomography angiography of the abdomen/pelvis was unremarkable for an acute process. His severe symptomatic hemolytic anemia was suspected from underlying G6PD deficiency and methemoglobinemia in the setting of recent nitrite use. His methemoglobinemia, being 1.5%, was lower than expected due to the delay in obtaining the arterial blood gas. Our patient met the criteria for treatment with methylene blue based on his severe symptoms and medical history. However, this was contraindicated in the presence of G6PD deficiency and the next-line treatment with ascorbic acid would also risk further hemolysis. In consultation with a hematology specialist, it was decided to continue conservative therapy. The patient’s lactic acid levels peaked at 11 mmol/L, which was attributed to end-organ damage from hypoperfusion secondary to severe hemolytic anemia. His hypotension and lactic acidosis resolved with volume resuscitation and hemodialysis. Subsequent arterial blood gas results showed a decrease in methemoglobin percentage and lactate acid.

The patient was successfully transferred from the intensive care unit to the general ward where he was eventually discharged home. Two months later, he followed up as an outpatient and his most recent hemoglobin was 10.6 g/dL and his total bilirubin was 1 mg/dl. 

## Discussion

Amyl nitrite-induced hemolytic anemia has been documented in both kinds of patients who have underlying anemia, particularly those with G6PD deficiency, and those without [1,3,9-15]. A prior in vitro experiment of a case and two controls of healthy volunteers whose blood sample was exposed to amyl nitrite resulted in Heinz bodies in all red blood cells, some hemolysis, and brown discoloration of the red blood cells, indicating methemoglobin formation in both the case and the controls [9]. This study also observed a dose-dependent relationship between amyl nitrite and hemolysis. This is an important observation, as we utilize nitrites in diagnostic modalities, and margin of safety is of importance [9]. Currently, it has been shown that 30 mL of amyl nitrite is needed to induce hemolytic anemia [11]. Hemolytic anemia secondary to inhaled amyl nitrite is treated with supportive care, however, methemoglobinemia is typically treated with intravenous methylene blue of 1-2 mg/kg over a five-minute period. The indication for methylene blue therapy is based on the absolute methemoglobin levels (>30%) or in symptomatic patients with any level [16]. In severe cases that do not respond to methylene blue therapy, other options include exchange transfusion and hyperbaric oxygen therapy [16]. Specifically, therapy for patients with G6PD deficiency differs, as they are unable to utilize methylene blue due to their low levels of NADPH [3]. Therapies for these patients include conservative therapy, exchange transfusion, or hyperbaric oxygen. This is important to note, as some hospitals are limited in resources and may not have the capabilities to provide such therapy. Along with acute therapy, preventative therapy is of utter importance. Currently, there is a lack of awareness amongst primary care providers concerning MSM patients and their use of poppers. The use of inhaled nitrites has been associated with an increased risk of HIV in the MSM population [17-19]. The risk of HIV transmission can increase threefold in the setting of nitrites use, as it increases the rate of unprotected sexual encounters [20]. Finally, it is important to note that a study revealed that up to 55.4% of patients who have used inhaled nitrites did not know that it carried the potential for side effects and an increased risk of HIV transmission due to risky sexual behaviors [19-20]. It can be considered a public health effort to increase awareness of nitrite use in the MSM amongst providers for proper counseling and for more targeted questions to this population, as literature has shown that MSM are less likely to divulge this kind of information.

## Conclusions

Inhaled nitrites have been a substance of recreational use for centuries, primarily among men who have sex with men (MSM). However, there is a lack of awareness of the use of inhaled nitrites in this population and the possible complications and health disparities it carries. This case report highlights the use of inhaled nitrites in an MSM male with an emphasis on the complications induced by this product, including severe hemolytic anemia and methemoglobinemia.

This report presents a unique scenario in which a glucose-6 phosphate dehydrogenase (G6PD)-deficient patient presented with hemolytic anemia and methemoglobinemia after recreational use of inhaled nitrites. The case was complicated with the management challenge of methemoglobinemia in a patient with G6PD deficiency. Prior literature has shown that the use of nitrites is a predictor of high-risk sexual behavior and is associated with positive human immunodeficiency virus (HIV) status. Increasing provider awareness for appropriate counseling is of importance.
